# Environmental Impacts of Large-Scale Spirulina (*Arthrospira platensis*) Production in Hellisheidi Geothermal Park Iceland: Life Cycle Assessment

**DOI:** 10.1007/s10126-022-10162-8

**Published:** 2022-09-07

**Authors:** Asaf Tzachor, Asger Smidt-Jensen, Alfons Ramel, Margrét Geirsdóttir

**Affiliations:** 1 Global Food Security Research Center & Centre for the Study of Existential Risk, University of Cambridge, Cambridge, UK; 2 School of Sustainability, Reichman University, Herzliya, Israel; 3 Centre for Food Technology, Danish Technological Institute (DTI), Århus, Midtjylland, Danmark; 4 Bioactive Compounds Group, Matís, Reykjavík, Iceland

**Keywords:** Spirulina, Photobioreactors, Life cycle assessment, Environmental impact, Carbon neutrality, Alternative protein

## Abstract

Spirulina algae (*Spirulina platensis*) cultivated in geothermally powered photobioreactors is here proposed as a potentially resource efficient, zero-carbon, and nutritious alternative to conventional beef meat. Employing a standard life cycle assessment, environmental impacts of large-scale Spirulina production in this facility are calculated. The production facility is sited in Orka náttúrunnar (ON Power) Geothermal Park, Iceland, and benefits from resource streams accessible through Hellisheiði (Hellisheidi) power station, including renewable electricity for illumination and power usage, hot and cold water streams for thermal management, freshwater for cultivation, and CO_2_ for biofixation. During cultivation, GHG-intensive ammonia-based fertilizers are replaced with macronutrients sourced from natural open mines. LCA results show that production of 1 kg of wet edible biomass in this facility requires 0.0378 m^2^ non-arable land, 8.36 m^3^ fresh water and is carbon neutral with − 0.008 CO_2_-eq GHG emissions (net zero). Compared with conventionally produced meat from beef cattle, Spirulina algae cultured in the ON Power Geothermal Park, referred to in this study as GeoSpirulina, requires less than 1% land and water and emits less than 1% GHGs. Considering food and nutritional security concerns, cultivation in a controlled environment agriculture system assures consistent nutritional profile year-round. Moreover, GeoSpirulina biomass assessed in this study contains all essential amino acids as well as essential vitamins and minerals. While keeping a balanced nutrition, for every kg beef meat replaced with one kg GeoSpirulina, the average consumer can save ~ 100 kg CO_2_-eq GHGs. It is concluded that environmental impacts of GeoSpirulina production in the Hellisheidi facility are considerably lower than those of conventionally produced ruminants.

**Graphical abstract:**

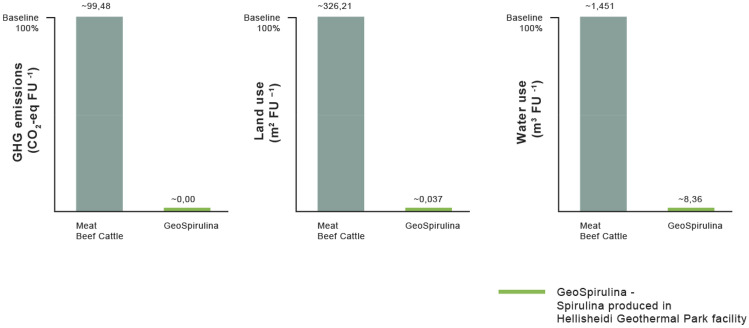

## Introduction

The nutritive role of ruminant meat in human diets is substantial. It is a source of essential amino and fatty acids, vitamins, and minerals. Beef meat is also an important nutritional source for bioavailable Iron (Fe). For this nutritional profile, meat has played a fundamental part in human species evolution (Pereira and Vicente 2013).

However, contemporary livestock production is a prime contributor to global greenhouse gas (GHG) emissions (CO_2_, CH_4_, and N_2_O) accounting for 18% of the global total. About a quarter of these emissions emanate from enteric fermentation of ruminants and release of methane (belching), and 31% of the emissions relate to manure management (Steinfeld et al. 2006; Tuomisto and Teixeira de Mattos 2011). In addition, meat production is a driver of land appropriation, including deforestation and land use change for the provision of pastures (de Oliveira Silva et al. 2016) and production of feedstocks (Nepstad et al. 2014). Furthermore, ruminant meat production accounts for 8% of global freshwater use (Steinfeld et al. 2006).

In consideration of the environmental ramifications of livestock production, and with future consumption of meat projected to rise by 1.7% in the period up to 2030 and by 1% in the following decades 2030–2050 (Henchion et al. 2014; Mathijs 2015), an increasing number of studies suggest a dietary transition to meat alternatives with similar or improved functional properties and substantially decreased environmental impacts (Tuomisto and Teixeira de Mattos 2011; Moomaw et al. 2017; Parodi et al. 2018; McClements 2020; Tzachor et al. 2021a, b; Barzee et al. 2021; Munialo et al. 2022; Tzachor 2022; Humpenöder et al. 2022).

One proposed alternative to meat has been the cultivation of Spirulina blue-green algae (*Arthrospira platensis*). Spirulina is a free-floating, microscopic, filamentous cyanobacterium, safe for human consumption, with recorded nutritional and potential therapeutic benefits, including antioxidant, anti-cancer, and anti-viral effects (Karkos et al. 2011). It is often considered an alternative food source to conventional foods by dint of its fast growth rate as a unicellular autotroph organism (Tzachor et al. 2021a, b), rich nutritional profile, and lack of a cellulose cell wall making it easily digestible (Marles et al. 2011; Karkos et al. 2011; Chen et al. 2016). Dietarily, Spirulina contains a large array of essential macro- and micronutrients available in meat. Previous analyses (Parodi et al. 2018) showed a higher content of protein (up to 70%) per 100 g than beef, as well as higher contents of various nutrients than beef (calculated per 1 g protein), including calcium (Ca), iron (Iron, Fe), and provitamin A (*β*-carotene).

For over five decades, Spirulina has been successfully cultivated in several techniques and apparatuses, in a range of geographical environments, including in open and closed systems, such as outdoor raceway ponds, and indoor tubular, vertical column, or flat-plate photobioreactors (PBRs) (Torzillo et al. 1986; Tredici 2004). Cultivation conditions, and the use of controlled environment agriculture (CEA) methods, have had a decisive significance in terms of biomass productivity, yield consistency, produce quality, biomass nutritional composition, organoleptic properties, production costs, and the environmental impact of biomass (Delrue et al. 2017). For example, in closed Spirulina PBRs, only a fraction of freshwater is lost by evaporation, with consequences for production inputs, expenses, and the environment (Torzillo et al. 1986).

Closed Spirulina PBRs are further characterized by low pathogen contamination risks, low space requirements, and minimal land footprint if situated on marginal, non-arable lands, high biomass concentration, and little-to-no dependence on climatic or weather conditions (Pulz 2001; Suh and Lee 2003; Tzachor 2019). Recent analyses delineated advanced configurations in which Spirulina is cultured in closed PBRs irradiated by light-emitting diodes (LED) where high photosynthetic photon flux at optimized wavelengths can be achieve to enhance photosynthesis efficiency (Nwoba et al. 2019). Such novel PBRs maintain steady internal physical, chemical, and biological conditions and therefore consistent and efficient performance (Tzachor et al. 2021a, b). Indeed, reliable, high-yield production is essential for a viable meat alternative.

To operate steadily, at low resource and GHG intensities, an indoor, large-scale, high-density culture system would require a constant stream of CO_2_ to meet carbon demand; nutrient supplies with low-carbon footprint, specifically nitrogen fertilizer, phosphorus fertilizer, and iron sulfate; hot and cold water streams for thermal management; freshwater for cultivation; residual heat for downstream processing; and a renewable non-intermittent energy source for so-called clean electricity, primarily for artificial illumination of culture.

Based on best available knowledge, currently only one large-scale, commercially viable configuration exists that meets these qualifications, situated at Hellisheiði (Hellisheidi) geothermal power park, near Reykjavík, Iceland.

The Hellisheidi Spirulina facility has an installed production capacity of 150,000 kg of edible wet biomass per year. The facility has previously participated in research funded by the European Institute of Innovation and Technology (EIT), a body of the European Union, under Horizon 2020, the EU Framework Program for Research and Innovation (Tzachor et al. 2021a, b). 

The ability to access Reykjavík Energy (Orkuveita Reykjavíkur), the utility company overseeing Hellisheidi geothermal park, the actual park, the Spirulina production facility, and the facility’s mass and energy balances, engineering design, and bill of materials, as well as conducting in situ analysis provided the rationale and motivation to undertake this study.

Furthermore, recent noteworthy studies that attempted to perform an LCA in the vein of this research have either analyzed Chlorella (*Chlorella vulgaris*) production in open raceway ponds (Yadav et al. 2020), Spirulina production in open raceway ponds (Ye et al. 2018) or have analyzed modeled production in open and closed systems—not actual (Smetana et al. 2017; Rodríguez et al. 2018; Quintero et al. 2021). An apparent gap in literature assessing Spirulina cultivated in PBRs irradiated by LED and integrated within a geothermal power complex with multiple available resource streams has bolstered the drive to carry out this research.

The purpose of this article is twofold. First, it aims to estimate the land use, water use, energy use, and GHG emissions for industrial-scale, geothermally powered indoor production of Spirulina. The second aim of this study is to establish the nutritional equivalence to beef meat and compare the environmental impacts of large-scale Spirulina production with conventionally produced meat from beef cattle.

## Methods and Materials

### System Boundaries and Production Process

For the calculation of environmental impacts, standard life cycle assessment (LCA) research method was used, based on guidelines and requirements delineated in ISO 14044:2006 – Environmental management – Life cycle assessment, reviewed and confirmed in 2022 (International Standard 2006).

All information regarding the cultivation system and process, from inoculation up-to facility gates, was obtained from the Hellisheidi Spirulina facility in Hellisheidi geothermal park, located in the region of Hengill, South West Iceland, N64°2′12″ W21°23′53″.

In the facility, Spirulina (*Arthrospira platensis*, UTEX 3086) is cultivated in modified Zarrouk medium (Rajasekaran et al. 2016). Cultivation is conducted in modular production units; each consists of a bundle of 80, 180-L flat panel airlift PBRs. Culture is kept under agitation pneumatically induced by CO2-enriched air, with flow aeration of 0.5 vvm (air volume/medium volume/minute). Temperatures are maintained at 31 ± 2 °C. pH is kept at 10.8 ± 0.2. Culture in PBRs is grown under red/blue/UV illumination (USP # 63/026,764) at 3.5 W/l with maximum irradiance of 750 μmol/(m^2^s).

**Fig. 1:**
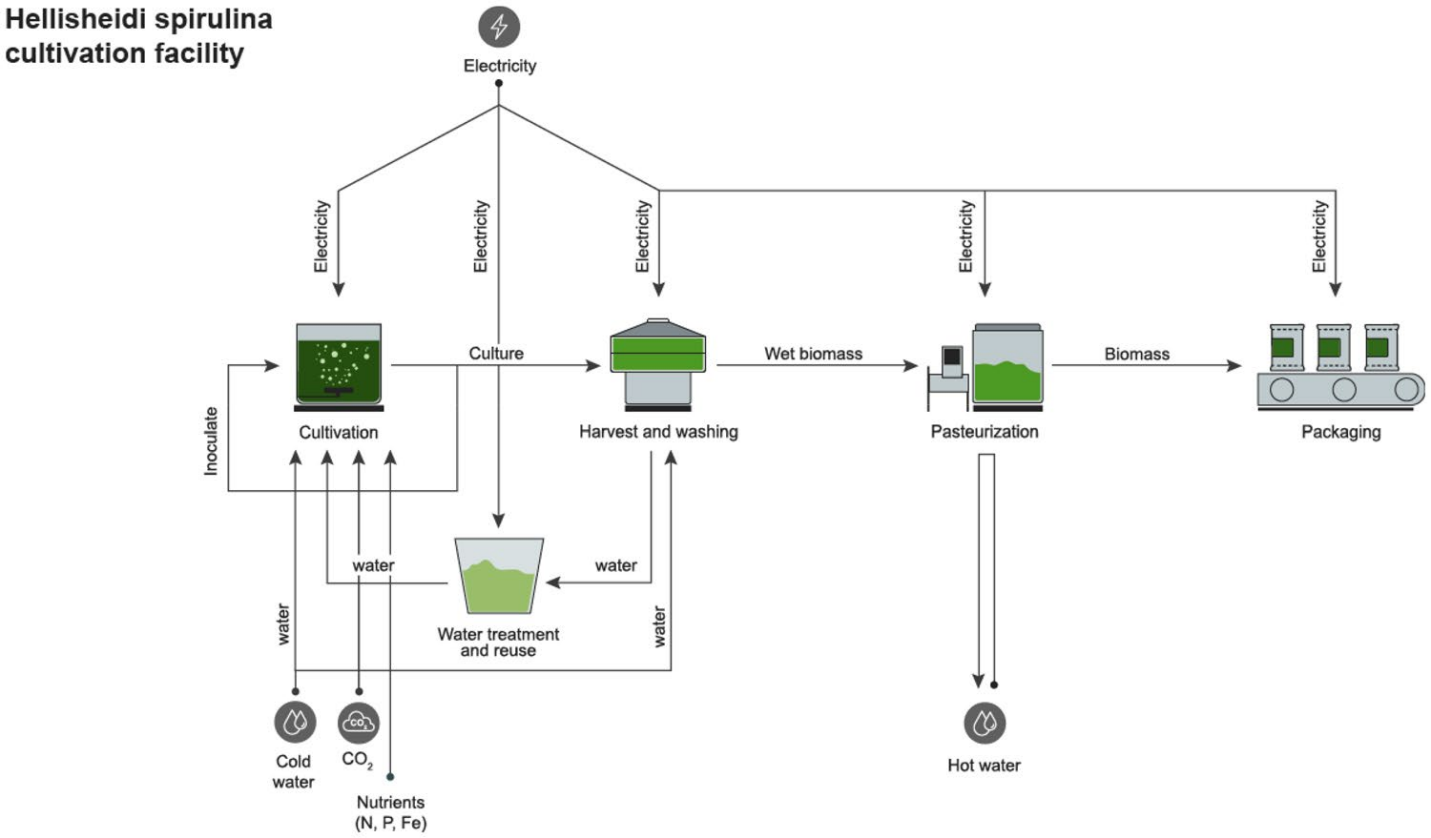
System diagram of Spirulina production and biomass flows

Light emitting diodes (LED, manufactured by Cree LED USA) are used for artificial illumination allowing spectral control of light and augmentation of photosynthesis (Schulze et al. 2014; Ooms et al. 2016). Considering energy-to-light LED efficiency and light-to-algae-biomass conversion efficiency, it was previously calculated that the energy required per Spirulina algal biomass is 143 kWh per 1 kg of ash free dry weight (AFDW) (Ooms et al. 2016). This figure corresponds the data obtained from the Hellisheidi Spirulina PBR facility. Moreover, approximately 50% of the electrical energy converts into light, with the remaining energy converted into heat (Ooms et al. 2016). In the production facility, residual heat is removed by liquid–liquid heat exchange using geothermal cooling water. Cooling pass-through water is provided by Hellisheidi geothermal park, alongside a stream of geothermal CO_2_ for biofixation in the culture (see Fig. 1).

During a continuous daily harvest, approximately 15% of the culture in each production unit is transferred to a rotation sieve set for harvest, washing, and downstream processing (micro- and ultrafiltration membranes). Residual medium water is treated on site and recycled back to cultivation (using micro- and ultrafiltration membranes). After washing, wet biomass is transferred to pasteurization. Hot geothermal waste stream is used as a heat source. After the pasteurization and cooling cycle, wet biomass is being parceled and packaged on site before it leaves factory gates.

Electricity for illumination, liquid pumping, gas blowing, culture harvesting and washing, water treatment, and packaging and clean-in-place (CIP) is supplied by a single source, an electric direct connection to Hellisheidi geothermal power station. Cold and hot water streams used in the facility are integrated within Hellisheidi geothermal plant streams. Transportation inside facility gates are negligible and do not count toward the LCA.

In terms of construction materials, a facility producing 150,000 kg of edible wet Spirulina per year, including all production phases up-to facility gates, is made from stainless steel, galvanized steel, carbon steel, aluminum, fiberglass, silicone, polypropylene, viton, polyethylene, and PVC-U and includes LED systems. The energy requirements and CO_2_-eq emissions for construction are calculated based on the facility bill of materials, in the following. Production process details are presented in Fig. 1.

**Fig. 2:**
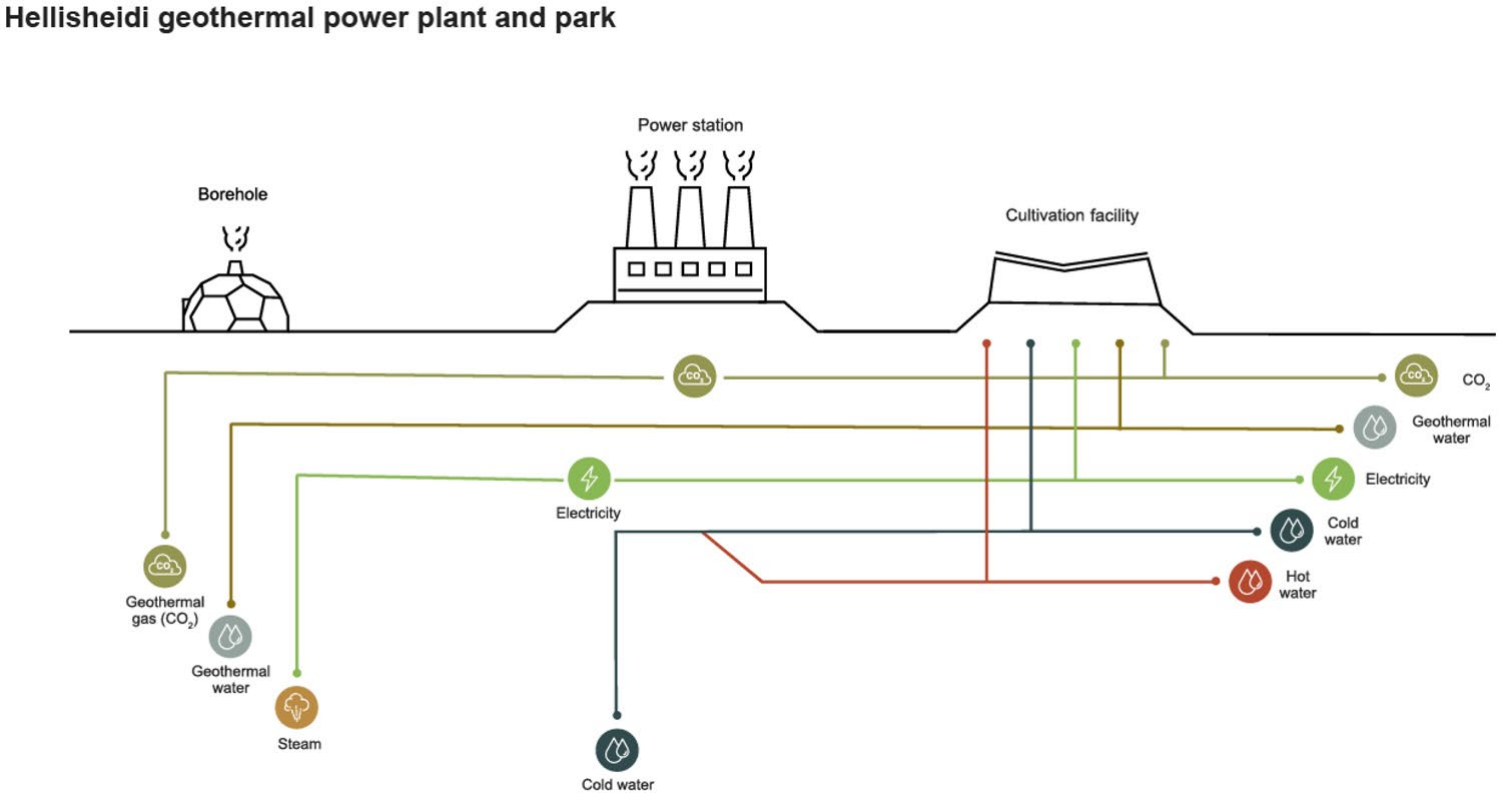
Hellisheidi geothermal park and resource streams including resources used as inputs in the Spirulina production facility. Based on in situ analysis and on Orka náttúrunnar (ON Power) Geothermal Park Reykjavík (Orka náttúrunnar 2022)

Resource streams in Hellisheidi geothermal park are presented in Fig. 2.

The functional unit (FU), toward which all environmental impacts are allocated, is 1 kg of edible Spirulina biomass (i.e., wet biomass with 61% water content) cultivated in the geothermal park. In this analysis, this Spirulina is referred to as “GeoSpirulina” in tables and figures. Laboratory analysis (conducted by Eurofins Scientific SE) shows the main nutritional content of GeoSpirulina wet biomass in Table 1, highlighting water, protein, essential amino acids (EAAs), and iron, Fe, per 100 g, alongside the nutritional content of ground meat from beef cattle (USDA 2022).

**Table 1:** Comparison of nutritional content of GeoSpirulina wet biomass produced in Hellisheidi facility with ground meat from beef cattle

	**Spirulina wet biomass** **(g/100 g)**	**Meat from beef cattle, ground** **(g/100 g)**
Solids (including EAAs, protein, and Iron)	39.0	44.0
Water	61.0	56.0
EAAs (threonine, valine, isoleucine, leucine, phenylalanine, lysine, histidine, methionine, tryptophan)	10.2	10.2
Protein	27.2	25.8
Iron, Fe	0.03	0.02

In this research, system boundaries cover the processes from input production up-to factory gates. The cultivation process described here produces a wet paste of edible Spirulina biomass. Outside factory gates, algal paste may be consumed as whole food (raw) or serve as an ingredient in the preparation of other foods.

The land use category includes the land requirements for Spirulina cultivation in this particular site, including cultivation, downstream processing, and miscellaneous needs (e.g., maintenance, storage). Indirect land use associated with the production of carbon and non-carbon inputs for the production units, namely the land used for geothermal energy production, CO_2_ stream for biofixation, and hot and cold water streams, are not included in this study because these have pre-existed to algal cultivation, remain under-utilized, and otherwise would have been wasted or emitted to the environment. Moreover, there are no competing demands for lands in Hengill area, for agricultural purposes or otherwise, as described in the following.

Decommissioning of the facility is not included in the calculation as construction materials would be possible to salvage, recycle, and reuse.

The LCA further accounts for the GHG emissions of producing nitrogen fertilizer, phosphorus fertilizer, and iron sulfate, used as nutrients for Spirulina production at ratios of 0.44, 1.26, and 0.16 kg FU^−1^, respectively (Papadaki et al. 2017), as well as for the production of a cleaning agent (Lye) for CIP of the production system.

## Data

### Allocations

The production of animal source foods, including beef meat, often cover several co-production processes to provide a variety of goods which may include meat cuts, fat, bones, and hide (i.e., leather from cattle). In LCAs, co-production processes require allocation of environmental impacts between different by-products (Nijdam et al. 2012). Considering that the Hellisheidi Spirulina production facility is a manufacturing system with just one stream of products (edible biomass in the form of wet paste), the environmental impacts in this study need only to account for this output. Therefore, allocations and sensitivity analyses are not included in this research.

### Land Inputs

An environmental impact assessment conducted by the European Investment Bank for Hellisheidi power plant determined that geothermal power production in this region has negligible effect on land and ecosystems, flora, fauna, and biota of hot springs, water resources, air quality, and cultural remains, residential, transport, or agricultural development. Lands in Hengill area are marginal and moss-covered with little-to-no vegetation. Animal life is scarce. Moreover, land and climate conditions make the region unsuitable for crop cultivation (EIB 2008). This assessment applies to all facilities situated in the geothermal park, including Hellisheidi Spirulina integrated production facility; therefore, the land use change value is zero.

In terms of direct, though non-arable land resources, 0.0378 m^2^ are required to produce 1 kg edible biomass year ^–1^. Table 2 shows land allocation by production phases.

**Table 2:** Land footprint of Hellisheidi Spirulina PBR facility per 1 kg biomass year ^–1^

**Production phase**	**Production machinery**	**Land footprint** **m**^**2**^**/(metric) kg year**^**−1**^
Cultivation	Production units	0.0292
Downstream processing	Harvest and washing; water recycling; pasteurization; packaging	0.0033
Storage	Biomass storage inside facility gates	0.0026
Others	Miscellaneous	0.0026
**Total**		**0.0378**

### Water Inputs

In accounting for freshwater uses, this study relies on a common framework (Milà i Canals et al. 2009) distinguishing between blue water (i.e., groundwater and surface), green water (i.e., rainwater), and grey water (i.e., water assimilating pollutants), used directly and indirectly in production processes.

Considering the production process analyzed in this research, surface and rainwater were excluded from the calculation, as well as grey water, since freshwater are mostly recycled with only a negligible volume (0.015 m^3^ kg^−1^), defined here as “waste,” is generated consisting of biomass and nutrient residuals, with no toxins and no runoff.

Similar to land uses in the geothermal park, streams of hot and cold freshwater from groundwater sources are already used for industrial purposes. The Spirulina PBR facility benefits from these resource streams, which otherwise would have remained under-utilized.

In addition, influences of algal cultivation and production on total annual water production in the Hellisheidi site, in terms of thermal management (liquid–liquid heat exchange, using water in the facility for cooling and heating), are insignificant taking into account the ~ 127.5 million m^3^ annual hot and cold water production of the power station and geothermal park (Reykjavík Energy 2020). Therefore, water circulated for cooling (for heat removal) and heating (during pasteurization) does not count toward the LCA.

As showed in Fig. 1, freshwater is used for cultivation, primarily as culture medium, and for washing of biomass. The system suffers no water losses. Table 3 provides freshwater use figures in the production of biomass.

**Table 3:** Water footprint of Hellisheidi integrated Spirulina production facility per 1 kg edible biomass

**Production phase**	**Water type**	**Water footprint** **m**^**3**^**/(liter) kg**^**−1**^
Cultivation	Blue	5.320
Washing (cleaning)	Blue	2.280
Others (miscellaneous)	Blue	0.760
**Total**		8.360

### Energy Inputs and GHG Emissions

Energy inputs for facility operations and GHG emissions for facility operations, construction materials of the facility, and nutrient production are calculated for one FU (1 kg edible Spirulina biomass). Primary energy includes both renewable (i.e., geothermal) and nonrenewable energy sources. GHG emissions were assessed as global warming potential by using the conventional 100 years’ time scale (GWP100).

Data for production operations were retrieved from publicly available analyses (2020) issued by Reykjavík Energy (Orkuveita Reykjavíkur) (Reykjavík Energy 2020), the utility operating Hellisheidi geothermal power station and park. Data for amounts and GHG intensities of construction materials were based on the facility’s bill of material and database retrieved from Mannvit Engineering, Iceland. Previously calculated GHG emissions of production of N fertilizer (Marques et al. 2009; Bäuerle 2017), P fertilizer (Randall et al. 2016; Chen et al. 2018), and iron sulfate (Randall et al. 2016) were used in this study. N and P fertilizers used in the Hellisheidi PBR facility are sourced from open-pit mines and are not based on energy intensive ammonia-based fertilizers.

The carbon fixed by Spirulina cells during cultivation contributes toward the LCA and is regarded as negative (− 0.702 kg CO_2_-eq kg^−1^ biomass), as otherwise it would have been released into the atmosphere. For this reason, it offsets positive CO_2_ emissions relating to the production process.

Energy inputs and GHG emissions are presented in Table 4.

**Table 4:** Energy inputs and GHG emissions of Hellisheidi integrated Spirulina production facility required to produce 1 kg edible biomass

**Construction materials**			**CO**_**2**_**-eq kg**^**−1**^
Fiberglass			0.051
Stainless steel			0.021
Galvanized steel			0.013
Carbon steel			0.008
Polypropylene			0.001
Viton			0.002
Polyethylene			0.001
Aluminum			0.037
LED systems			0.055
PVC-U			0.012
Silicone			0.001
**Total**			0.201
**Energy consumption**	**Production phase**	**kWh/kg**	**CO**_**2**_**-eq kg**^**−1**^
Lights (LED)	Cultivation	122	0.395
Pumps	Cultivation and downstream processing	6	0.027
Blowers (air, CO_2_)	Cultivation	8	0.026
Harvesters	Harvest and washing	3	0.010
Water treatment (Microfiltration and UV radiation)	Water recycling	0.5	0.002
Others	Pasteurization and packaging	0.2	0.001
**Total energy consumption**			**0.460**
**Nutrient consumption**			**CO**_**2**_**-eq kg**^**−1**^
Nitrogen fertilizer			0.009
Phosphorus fertilizer			0.020
Iron sulfate			0.000
**Total**			**0.028**
**Cleaning agent consumption**			
**Lye**			0.004
**Carbon dioxide biofixation**		**kg/kg DW**	**CO**_**2**_**-eq kg**^**−1**^
CO_2_ uptake		−1.8	−0.702
**Total CO**_**2**_**-eq kg**^**−1**^ **balance**			** −0.008**

## Results

For calculations, *Microsoft Excel 2019* spreadsheet program was used. Overall, producing 1 kg Spirulina edible biomass in the geothermal park requires 0.0378 m^2^ of no-arable, marginal lands. This translates to 37.8 m^2^ for 1-ton edible wet biomass. Production of FU^−1^ also requires 8.360 m^3^ of freshwater and 54.48 kWh of electricity. The cultivation phase accounts for the majority of resources utilized, with 77.36% of land, 63.63% of freshwater, and 87% of energy use. Cultivation also accounts for 66.34% of GHG emissions.

### Spirulina and Beef Meat Comparison

In recent years, a large number of LCA studies of animal food products, including beef meat, have been published. Studies analyzed a variety of FUs (e.g., 1 kg protein), environmental impacts (e.g., land use, methane emissions), supply chain phases (e.g., farm phase), food sources (e.g., ruminant meat), and production systems (e.g., meadow grazing, indoor feeding) (Nijdam et al. 2012). Different data sources and LCA approaches have given different results and figures in terms of environmental impacts, namely GHG emissions and land use. For example, with 1 kg protein as the FU, a meta-analysis of LCAs (Nijdam et al. 2012), which has not considered freshwater uses, suggested an upper limit of 640 GHG kg CO_2_-eq kg^−1^ protein from beef (with an average of 342.5 GHG kg CO_2_-eq kg^−1^ protein) and an upper limit for land use of 2,100 m^2^ y kg^−1^ protein from beef (with an average of 1068.5 m^2^ y kg^−1^ protein).

This research relies on more conservative figures for comparing Spirulina with beef meat. Specifically, it draws on a landmark study (Poore and Nemecek 2018) for the three comparisons: GHG intensity, land use, and freshwater use.

Comparing GHG intensities, while the production of 1 kg of Spirulina edible biomass in the Hellisheidi system is a carbon neutral process (− 0.008 CO_2_-eq kg^−1^), and the production of 1 kg meat from beef cattle was estimated to be 99.48 CO_2_-eq kg^−1^. Consequently, by replacing 1 kg beef meat with 1 kg Spirulina, an average omnivore or flexitarian may save ~ 100 kg CO_2_-eq of GHG emissions.

In comparing the land use requirements to produce 1 kg Spirulina with that required to produce 1 kg beef meat, the former requires 0.0378 m^2^ of no-arable, marginal lands per 1 kg per year, while the latter was estimated to require an average of 326.21 m^2^ kg year^−1^.

**Fig. 3:**
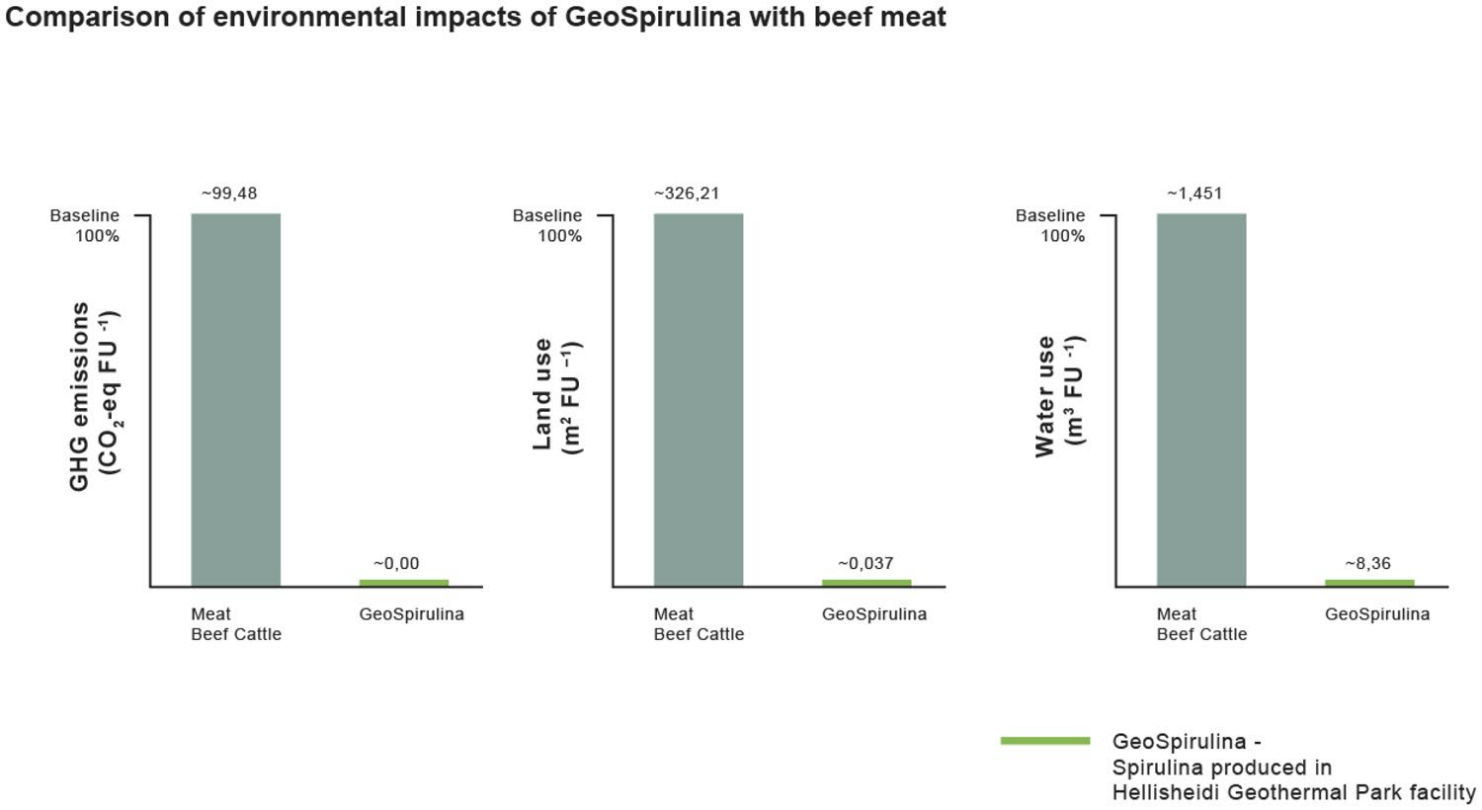
Comparison of GHG emissions (CO_2_-eq FU^−1^), land use (m^2^ FU^–1^) and water use (m^3^ FU^−1^) of GeoSpirulina produced in the Hellisheidi facility with conventionally produced meat from beef cattle, as a percent of the impacts of beef meat in each impact category. Values for GeoSpirulina are < 1% and therefore have a similar height in the bar chart

Comparing the freshwater footprint of the two products, Spirulina produced in the Hellisheidi system consumes 8.360 m^3^ kg^−1^, while beef meat requires an average of 1,451 m^3^ kg^−1^ (Fig. 3). Even if a more conservative estimate is used, 550 m^3^ for a single kg of beef (Chriki and Hocquette 2020), Hellisheidi produced Spirulina is still more than an order of magnitude more efficient.

## Discussion

Results show that Spirulina production in the Hellisheidi geothermally powered facility is carbon neutral (i.e., involves net-zero CO_2_ emissions), as its manufacture process balances between emitting CO_2_ and absorbing carbon. As opposed to these results, when PBRs rely on a non-renewable energy source, the GHG intensity of production may be significantly higher (Mata et al. 2018). In terms of achieving carbon neutrality, this is not an entirely original production process in the Icelandic context (Algalif 2022).

Since a stream of CO_2_ is necessary for photosynthesis and carbon fixation in autotrophic cell growth, the cultivation of Spirulina blue-green algae as a sustainable alternative to ruminant production is particularly attractive.

**Fig. 4:**
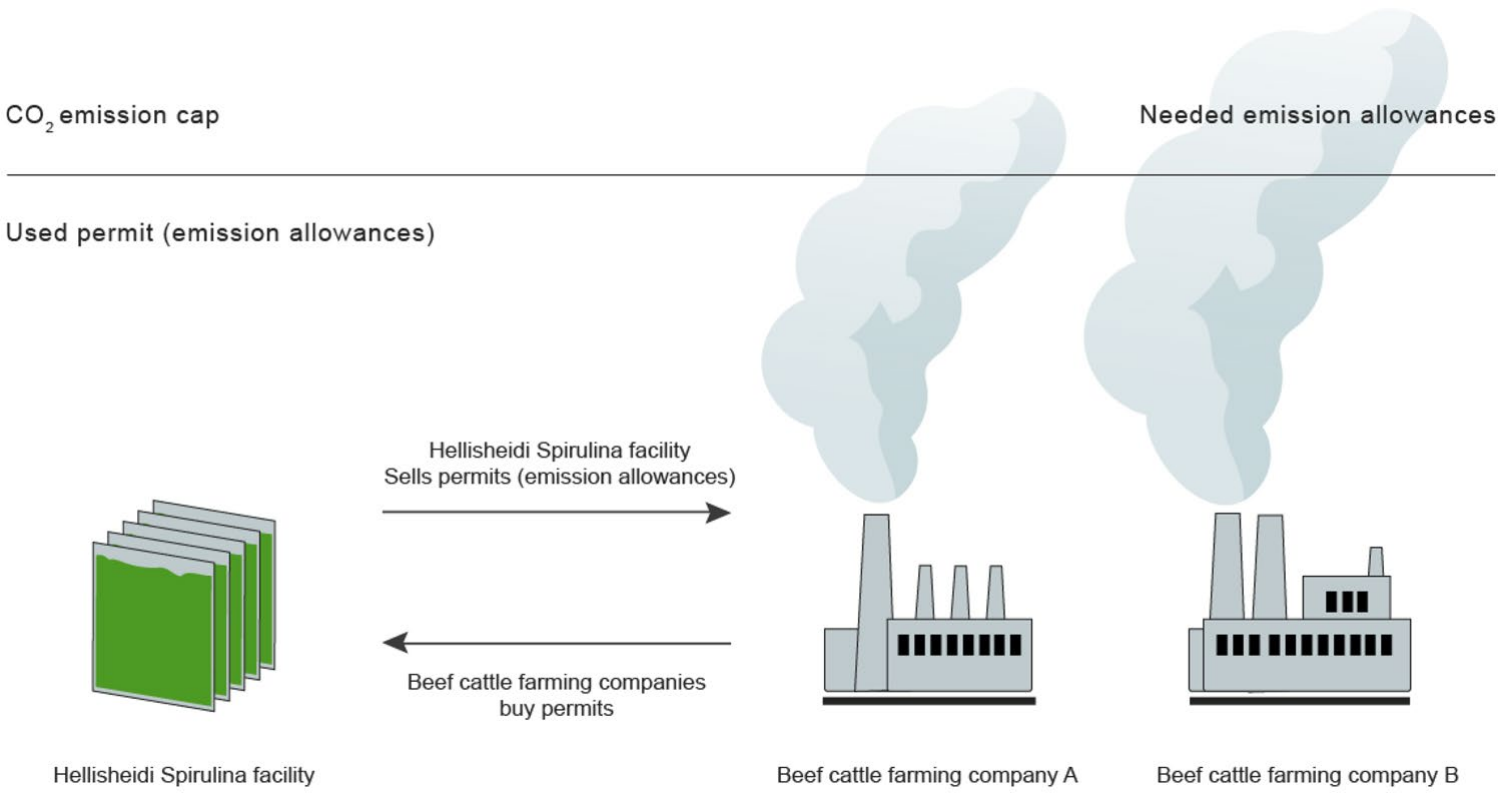
Illustration of potential carbon trading options in an emissions trading market system, based on analyses in this study

Advantageously, this configuration paves the way for direct decarbonization of food systems and diets by incorporating Spirulina from the geothermal park in meals as a beef meat replacement (including in omnivore, flexitarian, vegetarian, and vegan menus) and indirect decarbonization of food systems by means of issuing, selling, and purchasing carbon credits between enterprises (Fig. 4).

As opposed to other modeled studies based on assumptions, in this analysis land, water and energy input figures of Spirulina production are based on data retrieved from an actual large-scale operational facility and therefore suffer no uncertainty.

Moreover, Spirulina production in CEA techniques requires only a fraction of the land area that is used for producing the same amount of protein from conventionally produced beef. Consequently, even a gradual, incremental adoption of Spirulina as an alternative protein source in diets—to keep a balanced nutrition—can free lands currently used for grazing or feed-crops cultivation, for alternative uses such as reforestation.

Of course, if emissions related to land use change (LUC), that is land deforested and cleared for pasture grasses, hay, cereal and silage crops, and legumes grown specifically for animals, are allotted to beef cattle production, the total GHG emissions of conventional ruminant production would be higher than presented in this study (Nguyen et al. 2010; Flysjö et al. 2012), since the reference study used here for comparison excludes LUC. Accordingly, the carbon reduction potential of a so-called beef-to-Spirulina dietary shift would be higher.

### Ancillary Environmental Benefits

Incorporating Spirulina cultivated in geothermally powered PBRs in diets, as a ruminant meat substitute, even in small portions, could have potential benefits for conservation of flora and fauna, since deforestation driven by livestock production has been associated with wildlife habitat fragmentation and biodiversity loss (Montibeller et al. 2020).

Moreover, consuming proteins, EAAs, or iron from Spirulina cultivated in CEA systems would result in substantially lower nutrient losses to waterways, compared to conventionally produced ruminants, due to runoffs from livestock systems (Kato et al. 2009).

### Ancillary Dietary Benefits

Apart from environmental benefits, there are also potential nutritional benefits of substituting ruminant meat with Spirulina cultivated in PBRs. Spirulina contains a larger proportion of polyunsaturated fatty acids (PUFA) which according to previous studies have a positive effect on cardio-vascular diseases, atherosclerosis, coronary heart diseases, hyperlipidemia, and cancer (Otleş and Pire 2001). Furthermore, in addition to higher iron levels in Spirulina compared to beef (Table 1), the iron present in Spirulina is significantly more bioavailable (Puyfoulhoux et al. 2001). As the Spirulina is cultivated in CEA systems, a consistent nutritional profile could be achieved ensuring the described nutritional benefits.

### Ancillary Public Health Benefits

The substitution of meat from beef cattle with Spirulina cultivated in CEA systems might also result in a reduction in some of the adverse public health effects associated with livestock production. Livestock production uses large amounts of antibiotics resulting in antibiotic resistant strains of bacteria rendering bacterial infections difficult to treat (Alexander et al. 2008; Frieri et al. 2017). An ongoing reliance on ruminants could potentially increase the disease-burden and negatively impact public health (Sobur et al. 2019).

Additionally, cattle are responsible for a number of zoonotic diseases (McDaniel et al. 2014), the spread of which could potentially be mitigated with lower consumption of meat from ruminants by replacement with Spirulina produced in CEA systems. 

## Summary

The focus of this study was on the production of edible wet Spirulina biomass in a cultivation facility integrated in a geothermal power station and park, from inputs up-to factory gates, and, therefore, it does not account for environmental impacts throughout the cradle-to-grave supply chain, that is outside factory gates.

Nevertheless, the possibility to deploy similar modular facilities in a decentralized manner, dependent on necessary resource streams (e.g., renewable non-intermittent energy source, available CO_2_, available water streams for thermal management) yet independent of geographical conditions and otherwise limiting environmental factors (e.g., arable-land, favorable climate), promises to bring food production significantly closer to consumers than conventional animal source foods (Tzachor et al. 2021a, b), as in the case of remote locations such as Iceland. This would carry additional envisaged benefits such as increased food and protein self-sufficiency, reduced environmental impacts associated with shipping, and greater resilience of domestic food networks.

In the same vein, the modular property of the Spirulina production configuration analyzed in this research allows rapid and parallel production scale-up.

Alongside the development and deployment of large-scale Spirulina production systems, there is little concern regarding the consumer acceptance of Spirulina biomass. Spirulina is listed under the category Generally Recognized as Safe (GRAS) by the US Food and Drug Administration and is long purchased by consumers in various forms, from paste, to pills, to pressed powder. This puts Spirulina in a vantage comparing to other so-called sustainable beef meat replacements proposed in previous studies, such as insect larvae, that are atypical in different food cultures.

Furthermore, comparing the environmental impacts of Spirulina from the Hellisheidi facility with ruminant meat, this study concludes that consuming 1 kg of wet Spirulina instead of meat from beef cattle saves ~ 100 kg CO_2_-eq of GHGs, and over 1400 l of freshwater. This lower GHG intensity of protein production opens new options for trading in carbon permits (Fig. 4).

As aforementioned, the GHG intensities calculated are unique to the production configuration outlined in this study and are not necessarily inherent to Spirulina cultivation, as previous studies demonstrated.

Lastly, production in CEA techniques offers yield consistency that few agricultural systems, including those of beef cattle, can claim to achieve, especially under climate variability and alterations in temperatures and weather patterns. This should be essential for the future of food and protein security.

## Data Availability

Laboratory analysis of the nutritional content of Spirulina biomass used in Table 1, conducted by Eurofins Scientific SE Laboratory Testing, is available upon request. Other data and materials this study draws on are fully delineated in the text and reference list.

## References

[CR1] Alexander TW, Yanke LJ, Topp E, Olson ME, Read RR, Morck DW, McAllister TA (2008). Effect of subtherapeutic administration of antibiotics on the prevalence of antibiotic-resistant Escherichia coli bacteria in feedlot cattle. Appl Environ Microbiol.

[CR2] Algalif (2022) Carbon neutral certificate, CN20220110108, Algalif. Available at: https://algalif.is/wp-content/uploads/2022/01/LIF001-CarbonNeutral-product-CN20220110108-003.pdf (Retrieved August 14, 2022, from).

[CR3] Barzee TJ, Cao L, Pan Z, Zhang R (2021). Fungi for future foods. Journal of Future Foods.

[CR4] Bäuerle Y (2017) Thermal energy storage systems using recycled steel industry waste for concentrated solar power plants. Master of Science (M.Sc.)

[CR5] Chen W, Geng Y, Hong J, Yang D, Ma X (2018). Life cycle assessment of potash fertilizer production in China. Resour Conserv Recycl.

[CR6] Chen YH, Chang GK, Kuo SM, Huang SY, Hu I, Lo YL, Shih SR (2016). Well-tolerated Spirulina extract inhibits influenza virus replication and reduces virus-induced mortality. Sci Rep.

[CR7] Chriki S, Hocquette JF (2020) The myth of cultured meat: a review. Front Nutri 7 10.3389/fnut.2020.00007 PMC7020248 32118026

[CR8] de Oliveira Silva R, Barioni LG, Hall JAJ, Folegatti Matsuura M, Zanett Albertini T, Fernandes FA, Moran D (2016). Increasing beef production could lower greenhouse gas emissions in Brazil if decoupled from deforestation. Nat Clim Chang.

[CR9] Delrue F, Alaux E, Moudjaoui L, Gaignard C, Fleury G, Perilhou A, Sassi JF (2017). Optimization of Arthrospira platensis (Spirulina) growth: from laboratory scale to pilot scale. Fermentation.

[CR10] European Investment Bank (2008) Hellisheiði power plant environmental impact assessments. Available at: http://eib.org/attachments/pipeline/20080135_nts1_en (last accessed, August 14, 2022)

[CR11] Flysjö A, Cederberg C, Henriksson M, Ledgard S (2012). The interaction between milk and beef production and emissions from land use change–critical considerations in life cycle assessment and carbon footprint studies of milk. J Clean Prod.

[CR12] Frieri M, Kumar K, Boutin A (2017). Antibiotic resistance. J Infect Public Health.

[CR13] Henchion M, McCarthy M, Resconi VC, Troy D (2014). Meat consumption: trends and quality matters. Meat Sci.

[CR14] Humpenöder F, Bodirsky BL, Weindl I, Lotze-Campen H, Linder T, Popp A (2022). Projected environmental benefits of replacing beef with microbial protein. Nature.

[CR15] International Standard (2006) ISO 14044: 2006. Environmental management. Life cycle assessment. Requirements and guidelines

[CR16] Kato T, Kuroda H, Nakasone H (2009). Runoff characteristics of nutrients from an agricultural watershed with intensive livestock production. J Hydrol.

[CR17] Karkos PD, Leong SC, Karkos CD, Sivaji N, Assimakopoulos DA (2011) Spirulina in clinical practice: evidence-based human applications. Evidence-based complementary and alternative medicine

[CR18] Marles RJ, Barrett ML, Barnes J, Chavez ML, Gardiner P, Ko R, Griffiths J (2011). United States pharmacopeia safety evaluation of Spirulina. Crit Rev Food Sci Nutr.

[CR19] Marques A, Teixeira R, Lorena A, del Pino V, del Valle-Inclan Y, Navalho J, Domingos T (2009) Sustainability assessment of traditional solar salt. In 2nd International Conference on the Ecological Importance of Solar Saltworks (CEISSA 2009), Merida, Yucatan, Mexico 26–29

[CR20] Mata TM, Cameira M, Marques F, Santos E, Badenes S, Costa L, Martins AA (2018). Carbon footprint of microalgae production in photobioreactor. Energy Procedia.

[CR21] Mathijs E (2015). Exploring future patterns of meat consumption. Meat Sci.

[CR22] McClements DJ (2020). Future foods: is it possible to design a healthier and more sustainable food supply?. Nutr Bull.

[CR23] McDaniel CJ, Cardwell DM, Moeller RB, Gray GC (2014). Humans and cattle: a review of bovine zoonoses. Vector-Borne and Zoonotic Diseases.

[CR24] Milà i Canals, L., Chenoweth, J., Chapagain, A., Orr, S., Antón, A., & Clift, R.  (2009). Assessing freshwater use impacts in LCA: part I—inventory modelling and characterisation factors for the main impact pathways. The International Journal of Life Cycle Assessment.

[CR25] Montibeller B, Kmoch A, Virro H, Mander Ü, Uuemaa E (2020). Increasing fragmentation of forest cover in Brazil’s Legal Amazon from 2001 to 2017. Sci Rep.

[CR26] Moomaw W, Berzin I, Tzachor A (2017). Cutting out the middle fish: marine microalgae as the next sustainable omega-3 fatty acids and protein source. Ind Biotechnol.

[CR27] Munialo CD, Stewart D, Campbell L, Euston SR (2022) Extraction, characterisation and functional applications of sustainable alternative protein sources for future foods: A Review. Future Foods 100152

[CR28] Nepstad D, McGrath D, Stickler C, Alencar A, Azevedo A, Swette B, Hess L (2014) Slowing Amazon deforestation through public policy and interventions in beef and soy supply chains. Science 344(6188):1118–1123 10.1126/science.1248525 24904156

[CR29] Nguyen TLT, Hermansen JE, Mogensen L (2010). Environmental consequences of different beef production systems in the EU. J Clean Prod.

[CR30] Nijdam D, Rood T, Westhoek H (2012). The price of protein: review of land use and carbon footprints from life cycle assessments of animal food products and their substitutes. Food Policy.

[CR31] Nwoba EG, Parlevliet DA, Laird DW, Alameh K, Moheimani NR (2019). Light management technologies for increasing algal photobioreactor efficiency. Algal Res.

[CR32] Ooms MD, Dinh CT, Sargent EH, Sinton D (2016). Photon management for augmented photosynthesis. Nat Commun.

[CR33] Orka náttúrunnar (ON Power) (2022) Geothermal park and Hellisheiði power plant, Reykjavík. Available at: https://www.on.is/en/environment/geothermal-park/ (last accessed, 14 Aug 2022)

[CR34] Otleş S, Pire R (2001). Fatty acid composition of Chlorella and Spirulina microalgae species. J AOAC Int.

[CR35] Papadaki S, Kyriakopoulou K, Tzovenis I, Krokida M (2017). Environmental impact of phycocyanin recovery from Spirulina platensis cyanobacterium. Innov Food Sci Emerg Technol.

[CR36] Parodi A, Leip A, De Boer IJM, Slegers PM, Ziegler F, Temme EH, Van Zanten HHE (2018). The potential of future foods for sustainable and healthy diets. Nature Sustainability.

[CR37] Pereira PMDCC, Vicente AFDRB (2013). Meat nutritional composition and nutritive role in the human diet. Meat Sci.

[CR38] Poore J, Nemecek T (2018). Reducing food’s environmental impacts through producers and consumers. Science.

[CR39] Pulz O (2001). Photobioreactors: production systems for phototrophic microorganisms. Appl Microbiol Biotechnol.

[CR40] Puyfoulhoux G, Rouanet JM, Besançon P, Baroux B, Baccou JC, Caporiccio B (2001). Iron availability from iron-fortified spirulina by an in vitro digestion/Caco-2 cell culture model. J Agric Food Chem.

[CR41] Quintero CD, Ventura A, Lepine O, Pruvost J (2021). Eco-design of spirulina solar cultivation: key aspects to reduce environmental impacts using Life Cycle Assessment. J Clean Prod.

[CR42] Rajasekaran C, Ajeesh CM, Balaji S, Shalini M, Ramamoorthy SIVA, Ranjan DAS, Kalaivani T (2016). Effect of modified Zarrouk’s medium on growth of different Spirulina strains. Walailak Journal of Science and Technology (WJST).

[CR43] Randall P, Meyer D, Ingwersen W, Vineyard D, Bergmann M, Unger S, Gonzalez M (2016) Life cycle inventory (LCI) data-treatment chemicals, construction materials, transportation, on-site equipment, and other processes for use in spreadsheets for environmental footprint analysis (SEFA). EPA/600/R16/176, U.S. Environmental Protection Agency, Washington DC

[CR44] Reykjavík Energy (Orkuveita Reykjavíkur) (2020) Environmental data – Reykjavik Energy 2015–2020. Reykjavík Energy. Available at: https://annualreport2020.or.is/documents/612/EN_Environmental_data_of_the_OR_Group_2020.pdf (last accessed, 14 Aug 2022)

[CR45] Rodríguez R, Espada JJ, Moreno J, Vicente G, Bautista LF, Morales V, Dufour J (2018). Environmental analysis of Spirulina cultivation and biogas production using experimental and simulation approach. Renewable Energy.

[CR46] Schulze PS, Barreira LA, Pereira HG, Perales JA, Varela JC (2014). Light emitting diodes (LEDs) applied to microalgal production. Trends Biotechnol.

[CR47] Smetana S, Sandmann M, Rohn S, Pleissner D, Heinz V (2017). Autotrophic and heterotrophic microalgae and cyanobacteria cultivation for food and feed: life cycle assessment. Biores Technol.

[CR48] Sobur MA, Sabuj A, Sarker R, Rahman A, Kabir S, Rahman MT (2019) Antibiotic-resistant Escherichia coli and Salmonella spp. associated with dairy cattle and farm environment having public health significance. Veterinary World 12(7):984–993 10.14202/vetworld.2019.984-993 PMC6702575 31528022

[CR49] Steinfeld H, Gerber P, Wassenaar TD, Castel V, Rosales M, Rosales M, de Haan C (2006) Livestock’s long shadow: environmental issues and options. Food & Agriculture Org

[CR50] Suh IS, Lee CG (2003). Photobioreactor engineering: design and performance. Biotechnol Bioprocess Eng.

[CR51] Torzillo G, Pushparaj B, Bocci F, Balloni W, Materassi R, Florenzano G (1986). Production of Spirulina biomass in closed photobioreactors. Biomass.

[CR52] Tredici MR (2004). Mass production of microalgae: photobioreactors. Handbook of Microalgal Culture: Biotechnology and Applied Phycology.

[CR53] Tuomisto HL, Teixeira de Mattos MJ (2011). Environmental impacts of cultured meat production. Environ Sci Technol.

[CR54] Tzachor A (2019). The future of feed: integrating technologies to decouple feed production from environmental impacts. Ind Biotechnol.

[CR55] Tzachor A (2022). Novel foods for human and planetary health. Nature Food.

[CR56] Tzachor A, Richards CE, Holt L (2021). Future foods for risk-resilient diets. Nature Food.

[CR57] Tzachor A, Rozen O, Khatib S, Jensen S, Avni D (2021). Photosynthetically controlled spirulina, but not solar spirulina, inhibits TNF-α secretion: potential implications for COVID-19-related cytokine storm therapy. Mar Biotechnol.

[CR58] U.S. Department of Agriculture (2022) Beef products, beef, ground, 80% lean meat / 20% fat, patty, cooked, broiled, nutrients. USDA, Agricultural Research Service. Available at: https://fdc.nal.usda.gov/fdc-app.html#/food-details/171797/nutrients (last accessed, 14 Aug 2022).

[CR59] Yadav G, Dubey BK, Sen R (2020). A comparative life cycle assessment of microalgae production by CO2 sequestration from flue gas in outdoor raceway ponds under batch and semi-continuous regime. J Clean Prod.

[CR60] Ye C, Mu D, Horowitz N, Xue Z, Chen J, Xue M, Zhou W (2018). Life cycle assessment of industrial scale production of Spirulina tablets. Algal Res.

