# Correction to: Environmental Impacts of Large-Scale Spirulina (Arthrospira platensis) Production in Hellisheidi Geothermal Park Iceland: Life Cycle Assessment

**DOI:** 10.1007/s10126-022-10173-5

**Published:** 2022-10-12

**Authors:** Asaf Tzachor, Asger Smidt-Jensen, Alfons Ramel, Margrét Geirsdóttir

**Affiliations:** 1 Global Food Security Research Center & Centre for the Study of Existential Risk, University of Cambridge, Cambridge, UK; 2 School of Sustainability, Reichman University, Herzliya, Israel; 3 Centre for Food Technology, Danish Technological Institute (DTI), Århus, Midtjylland Danmark; 4 Bioactive Compounds Group, Matís, Reykjavík, Iceland

**Correction to: Marine Biotechnology** **https://doi.org/10.1007/s10126-022-10162-8**

The original online version of this article was revised: In the Results section, the sentence 'Production of FU ^–1^ also requires 8.360 m^3^ of freshwater and 137.9 kWh of electricity.', the unit '137.9 kWh' should instead be '54.48 kWh'.

The original article has been corrected.

